# ﻿New species and records of *Neomassaria*, *Oxydothis* and *Roussoella* (Pezizomycotina, Ascomycota) associated with palm and bamboo from China

**DOI:** 10.3897/mycokeys.93.89888

**Published:** 2022-11-10

**Authors:** Hong Min Hu, Li Li Liu, Xu Zhang, Yan Lin, Xiang Chun Shen, Si Han Long, Ji Chuan Kang, Nalin N. Wijayawardene, Qi Rui Li, Qing De Long

**Affiliations:** 1 State Key Laboratory of Functions and Applications of Medicinal Plants, Guizhou Medical University, Guiyang, China; 2 The Key Laboratory of Optimal Utilization of Natural Medicine Resources, School of Pharmaceutical Sciences, Guizhou Medical University, University Town, Guian New District, Guizhou, China; 3 Key Laboratory of Infectious Immune and Antibody Engineering of Guizhou Province, Cellular Immunotherapy Engineering Research Center of Guizhou Province, School of Biology and Engineering, Guizhou Medical University, Guiyang 550025, China; 4 Immune Cells and Antibody Engineering Research Center of Guizhou Province, School of Biology and Engineering, Guizhou Medical University, Guiyang 550025, China; 5 The Engineering and Research Center for Southwest Bio-Pharmaceutical Resources of National Education Ministry of China, Guizhou University, Guizhou, China; 6 Center for Yunnan Plateau Biological Resources Protection and Utilization, College of Biological Resource and Food Engineering, Qujing Normal University, Qujing, Yunnan 655011, China; 7 Section of Genetics, Institute for Research and Development in Health and Social Care No: 393/3, Lily Avenue, Off Robert Mawatha, Battaramulla 10120, Sri Lanka

**Keywords:** 2 new taxa, bambusicolous and palm fungi, phylogeny, Pleosporales, taxonomy, Xylariales

## Abstract

Several micro fungi were gathered from bamboo and palm in Guizhou Province, China. In morphology, these taxa resemble *Neomassaria*, *Roussoella* and *Oxydothis*. Multi-gene phylogenetic analyses based on combined ITS, LSU, SSU, *rpb*2 and *tef*1 loci confirmed that two are new geographical records for China, (*viz. Roussoellasiamensis*, *Neomassariafabacearum*), while two of them are new to science (*viz. Oxydothisfortunei* sp. nov. and *Roussoellabambusarum* sp. nov.). The stromata of *Roussoellabambusarum* are similar to those of *R.thailandica*, but its ascospores are larger. In addition, multi-gene phylogenetic analyses show that *Oxydothisfortunei* is closely related to *O.inaequalis*, but the J- ascus subapical ring as well as the ascospores of *O.inaequalis* are smaller. Morphological descriptions and illustrations of all species are provided.

## ﻿Introduction

Ascomycetous taxa on bamboo and palm are commonly observed with immersed ascomata (Dai et al. 2017). *Oxydothis* Penz. & Sacc. and *Roussoella* Sacc. are well-documented on bamboo and palms in different localities in Asia (Liu et al. 2014; Konta et al. 2016; Dai et al. 2017).

The family Oxydothidaceae S. Konta & K.D. Hyde was erected to accommodate a single genus (*Oxydothis*) by Konta et al. (2016). Species of *Oxydothis* are characterized by the cylindrical asci with a J+ (rarely J-) subapical apparatus and filiform to fusiform, hyaline,1-septate ascospores with spine-like or rounded ends (Konta et al. 2016). Anamorph is *Selenosporella* sp. (descriptions from Samuels and Rossman 1987). Eighty-five epithets of *Oxydothis* have been listed in Index Fungorum (accession date: 1 May 2022). *Oxydothis* species (such as *O.oraniopsidis* Fröhlich & Hyde, *O.cyrtostachicola* Hidayat, To-Anun & K.D. Hyde, *O.garethjonesii* Konta & Hyde) are the initial colonizers of dead palm material (Hyde 1993; Fröhlich and Hyde 1994; Hidayat et al. 2006; Konta et al. 2016).

Liu et al. (2014) introduced Roussoellaceae Jian K. Liu et al. to accommodate three genera, i.e. *Neoroussoella* Jian K. Liu et al., *Roussoella* Sacc. and *Roussoellopsis* I. Hino & Katum (Liu et al. 2014). Later, *Appendispora* K.D. Hyde, *Cytoplea* Bizz. & Sacc., *Elongatopedicellata* Jin F. Zhang et al., *Immotthia* M.E. Barr and, *Pararoussoella* Wanas et al., were added to this family (Hyde 1994; Hyde et al. 1996; Ariyawansa et al. 2015; Hyde et al. 2017; Phookamsak et al. 2019; Wijayawardene et al. 2020). Most species of Roussoellaceae were reported as saprophytic taxa on the terrestrial plants including bamboo, palms and mangroves (Liu et al. 2014; Jiang et al. 2019; Poli et al. 2020). The members of this family have 4–8 spored, and bitunicate asci with aseptate, brown to dark brown ascosporic, melanconiopsis-like or neomelanconium-like asexual morphs (Liu et al. 2014).

Hyde et al. (2016) introduced the monotypic genus *Neomassaria* Mapook et al. to accommodate *N.fabacearum* Mapooket et al. in Neomassariaceae. The *Neomassaria* is characterized by globose to subglobose ascomata with fusoid, hyaline, 1-septate ascospores, with or without a sheath but the asexual morph is undetermined (Hyde et al. 2016; Ariyawansa et al. 2018; Yang et al. 2022). Currently, only three species have been reported, *viz.*, *Neomassariafabacearum* from the branch of *Hippocrepisemerus* (L.) Lassen (Hyde et al. 2016), *N.formosana* H.A. Ariyaw. et al. on a dead stem of *Rhododendron* sp. (Ariyawansa et al. 2018), and *N.hongheensis* E.F. Yang & Tibpromma on a decayed branch of *Mangiferaindica* L. (Yang et al. 2022).

In this study, several specimens of bamboo and palm were collected from Guizhou Province. Based on their morphology and phylogeny, two new species and two new records from China are herein reported. Full descriptions, photo plates of macro-and micro-morphological characteristics and a phylogenetic tree to show the phylogenetic placement of the new records and the new species are provided.

## ﻿Materials and methods

### Fungi collections, isolations and morphology

From 2021 to 2022, fresh materials were collected from bamboo and palms in forests and nature reserves of Guizhou Province, China, and returned to the lab in paper or plastic bags. Samples were treated and examined with the method described by Taylor and Hyde (2003). Morphological characteristics were examined using a Nikon SMZ 745 series stereomicroscope and photographed using a Canon 700D digital camera. Melzer’s iodine reagent was used for testing the amyloid reaction of the apical apparatus structures. Micro-morphological structures were photographed using a Nikon digital camera (Canon 700D) fitted to a light microscope (Nikon Ni). At least 30 ascospores and asci of each specimen were measured using the Tarosoft image framework (v. 0.9.0.7). Photo plates were arranged and improved using Adobe Photoshop CS6 software. Specimens were kept in the Herbarium of Guizhou Medical University (**GMB**) and Herbarium of Kunming Institute of Botany, Chinese Academy of Sciences (**KUN-HKAS**).

Isolations were made by single spore isolation (Long et al. 2019) and germinated spores were transferred onto potato dextrose agar (PDA) medium for purification. The colonies grown on PDA at 25 °C were transferred to three 1.5 mL microcentrifuge tubes filled with sterile water and stored with 10% glycerol at –20 °C. Living cultures were deposited at Guizhou Medical University Culture Collection (GMBC).

### ﻿DNA extraction, Polymerase chain reaction (PCR) amplification and sequencing

The OMEGA E.Z.N.A. Fungal Genomic DNA Extraction Kit (D3390, Guangzhou Feiyang Bioengineering Co., Ltd, China) was used to extract genomic DNA from fresh fungal mycelium, according to the manufacturer’s instructions. The extracted DNA was stored at –20 °C.

ITS5/ITS4 (White et al. 1990), LR0R/LR5 (Vilgalys and Hester 1990) and NS1/NS4 primers (White et al. 1990) were used for the amplification of ITS, LSU and SSU. Translation elongation factor 1-α gene region (*tef*1) and RNA polymerase II second largest subunit (*rpb*2) genes were amplified using EF1-983F and EF 1-2218R (Rehner 2001), rpb2-5f and rpb2-7cr primers (Liu et al. 1999) respectively.

PCR was carried out in a volume of 25 μL containing 9.5 μL of ddH_2_O, 12.5 μL of 2× Tap PCR Master Mix (2× Tap Master Mix with dye, TIANGEN, China), 1 μL of DNA extracts and 1 μL of forward and reverse primers in each reaction. The PCR thermal cycle of ITS, LSU, SSU and *tef*1 amplification is as follows: initially 95 °C for 5 minutes, followed by 35 cycles of denaturation at 94 °C for 1 minute, annealing at 52 °C for 1 minute, elongation at 72 °C for 1.5 minutes, and final extension at 72 °C for 10 minutes. The PCR thermal cycle program for the partial *rpb*2 was followed as initially 95 °C for 5 minutes, followed by 35 cycles of denaturation at 95 °C for 1 minute, annealing at 54 °C for 2 minutes, elongation at 72 °C for 1.5 minutes, and final extension at 72 °C for 10 minutes. The amplified PCR fragments were sent to Sangon Biotech (Shanghai) Co., China, for sequencing. Generated new sequences of ITS, LSU, SSU, *rpb*2 and *tef*1 regions were deposited in GenBank (Table 1).

**Table 1.** Taxa of *Neomassaria*, *Roussoella*, *Oxydothis* and related genera used for phylogenetic analyses.

Species	Strain number	GenBank Accession number					References
		ITS	LSU	SSU	*rpb*2	*tef*1	
*Acrocordiellaocculta*	RS10	KT949894	NA	NA	NA	NA	Jaklitsch et al. (2016)
*Acrocordiellaocculta*	RS9	KT949893	NA	NA	NA	NA	Jaklitsch et al. (2016)
*Aigialusgrandis*	JK 5244A	NA	GU301793	GU296131	GU371762	NA	Schoch et al. (2009)
*Albertiniellapolyporicola*	CBS 457.88	NA	AF096185	AF096170	NA	NA	Suh et al. (1999)
*Amniculicolalignicola*	CBS 123094 (HT)	NA	EF493861	EF493863	EF493862	GU456278	Zhang et al. (2009)
*Amphibambusabambusicola*	MFLUCC 11-0617	KP744433	KP744474	NA	NA	NA	Liu et al. (2015)
*Amphisphaeriasorbi*	MFLUCC 13 C0721	NA	KP744475	NA	NA	NA	Liu et al. (2015)
*Amphisphaeriaumbrina*	AFTOL-ID 1229 (ET)	NA	FJ176863	FJ176809	NA	NA	Unpublished
*Apiosporabambusae*	ICMP 6889	NA	DQ368630	DQ368662	NA	NA	Tang et al. (2007)
*Apiosporahydei*	CBS 114990	KF144890	KF144936	NA	NA	NA	Crous et al. (2013)
*Apiosporamontagnei*	AFTOL-ID 951	NA	DQ471018	NA	NA	NA	Spatafora et al. (2006)
*Arecophilabambusae*	HKUCC 4794	NA	AF452038	AY083802	NA	NA	Jeewon et al. (2003)
*Arthopyreniasaltuensis*	CBS 368.94	KF443410	AY538339	NA	KF443397	KF443404	Lumbsch et al. (2005)
*Arthriniumphaeospermum*	HKUCC 3395	NA	AY083832	AY083816	NA	NA	Unpublished
*Astrosphaeriellaaggregata*	MAFF 239486 (HT)	NA	AB524591	AB524450	AB539092	AB539105	Tanaka et al. (2009)
*Bartaliniarobillardoides*	CBS 122705 (ET)	KJ710460	KJ710438	NA	NA	NA	Crous et al. (2014)
*Beltraniapseudorhombica*	CBS138003	KJ869158	KJ869215	NA	NA	NA	Crous et al. (2014)
*Beltraniellaendiandrae*	CBS137976	KJ869128	KJ869185	NA	NA	NA	Crous et al. (2014)
*Broomellavitalbae*	MFLUCC 15-0023	KP757755	KP757751	KP757759	NA	NA	Liu et al. (2015)
*Cainiagraminis*	CBS 136.62 (ET)	NA	AF431949	AF431948	NA	NA	Lumbsch et al. (2002)
*Cephalothecafoveolata*	UAMH11631 (ET)	KC408422	KC408398	NA	NA	NA	Unpublished
*Clypeosphaeriauniseptata*	HKUCC6349 (ET)	NA	DQ810219	DQ810255	NA	NA	Unpublished
*Colletotrichumgloeosporioides*	LC0555	JN943090	JN940412	JN940356	NA	NA	Schoch et al. (2012)
*Coniocessiaanandra*	Co108	GU553338	GU553349	NA	NA	NA	Asgari et al. (2011)
*Coniocessiamaxima*	Co117	GU553332	GU553344	NA	NA	NA	Asgari et al. (2011)
*Coniocessianodulisporioides*	Co126 (ET)	GU553333	GU553352	NA	NA	NA	Asgari et al. (2011)
*Cordanaabramovii*	PE 0063-1a	NA	KF83336	NA	NA	NA	Zelski et al. (2014)
*Cordanainaequalis*	CBS 508.83	HE672146	HE672157	NA	NA	NA	Unpublished
*Cordanapauciseptata*	CBS 121804 (ET)	HE672149	HE672160	NA	NA	NA	Unpublished
*Creosphaeriasassafras*	CM AT-018	NA	DQ840056	NA	NA	NA	Unpublished
*Cryptendoxylahypophloia*	WM10.89	NA	HQ014708	NA	NA	NA	Unpublished
*Cycasicolagoaensis*	MFLU 17-0581 (HT)	NR_157510	NG_059057	NA	NA	NA	Wanasinghe et al. (2018)
*Delitschiadidyma*	UME 31411	NA	DQ384090	AF242264	NA	NA	Kruys et al. (2006)
*Delitschiawinteri*	AFTOL-ID 1599	NA	DQ678077	DQ678026	DQ677975	DQ677922	Schoch et al. (2006)
*Diatrypedisciformis*	AFTOL-ID 927	NA	DQ470964	DQ471012	NA	NA	Spatafora et al. (2006)
*Diatrypepalmicola*	MFLUCC 11-0020	KP744438	KP744482	KP753950	NA	NA	Liu et al. (2015)
*Diatrypewhitmanensis*	ATCC MYA-4417	FJ746656	NA	NA	NA	NA	Unpublished
*Didymellaexigua*	CBS 183.55 (HT)	NA	NA	GU296147	GU371764	NA	Schoch et al. (2009)
*Eutypalata*	CBS 208.87	DQ006927	NA	NA	NA	NA	Rolshausen et al. (2006)
*Herpotrichiajuniperi*	AFTOL-ID 1608	NA	DQ678080	DQ678029	DQ677978	DQ677925	Schoch et al. (2006)
*Hyalotiellaspartii*	MFLUCC 13-0397	KP757756	KP757752	KP757760	NA	NA	Liu et al. (2015)
*Hyponectriabuxi*	UME 31430	NA	AY083834	AF130976	NA	NA	Unpublished
*Immersidiscosiaeucalypti*	HHUF 29920	AB594793	AB593722	AB593703	NA	NA	Tanaka et al. (2011)
*Iodosphaeriatongrenensis*	MFLU15-0393	KR095282	KR095283	KR095284	NA	NA	Li et al. (2015)
*Lepteutypacupressi*	IMI 052255	NA	AF382379	AY083813	NA	NA	Jeewon et al. (2002)
*Leptosphaerulinaaustralis*	CBS 317.83	NA	GU301830	GU296160	GU371790	GU349070	Schoch et al. (2009)
*Lopadostomaturgidum*	LT2	KC774618	NA	NA	NA	NA	Voglmayr et al. (2017)
*Lophiostomaarundinis*	AFTOL-ID 1606	NA	DQ782384	DQ782383	DQ782386	DQ782387	Schoch et al. (2006)
*Lophiostomamacrostomoides*	CBS 123097	NA	FJ795439	FJ795482	FJ795458	GU456277	Zhang et al. (2009)
*Massariaanomia*	WU 30509	NA	HQ599378	HQ599453	NA	HQ599318	Voglmayr et al. (2011)
*Massariaariae*	WU 30510 (HT)	NA	HQ599381	HQ599458	NA	HQ599321	Voglmayr et al. (2011)
*Massariaaucupariae*	WU 30512	NA	HQ599384	HQ599455	NA	HQ599324	Voglmayr et al. (2011)
*Massariacampestris*	WU 30610	NA	HQ599386	NA	NA	HQ599326	Voglmayr et al. (2011)
*Massariaconspurcata*	WU 30519	NA	HQ599393	HQ599441	NA	HQ599333	Voglmayr et al. (2011)
*Massariagigantispora*	WU 30521	NA	HQ599397	HQ599447	NA	HQ599337	Voglmayr et al. (2011)
*Massariainquinans*	WU 30527	NA	HQ599402	HQ599444	HQ599460	HQ599342	Voglmayr et al. (2011)
*Massarialantanae*	WU 30533 (HT)	NA	HQ599406	HQ599443	NA	HQ599346	Voglmayr et al. (2011)
*Massariamacra*	WU 30535 (HT)	NA	HQ599408	HQ599450	NA	HQ599348	Voglmayr et al. (2011)
*Massariamediterranea*	WU 30547 (HT)	NA	HQ599414	NA	NA	HQ599354	Voglmayr et al. (2011)
*Massariaparva*	WU 30553	NA	HQ599418	HQ599467	NA	NA	Voglmayr et al. (2011)
*Massariaplatanoidea*	WU 30556	NA	HQ599423	NA	NA	HQ599362	Voglmayr et al. (2011)
*Massariapyri*	WU 30562 (HT)	NA	HQ599424	HQ599445	NA	HQ599363	Voglmayr et al. (2011)
*Massariaulmi*	WU 30565	NA	HQ599427	NA	NA	HQ599366	Voglmayr et al. (2011)
*Massariavindobonensis*	WU 30602	NA	HQ599432	NA	NA	HQ599371	Voglmayr et al. (2011)
*Massariavomitoria*	WU 30606	NA	HQ599437	HQ599440	HQ599466	HQ599375	Voglmayr et al. (2011)
*Massariazanthoxyli*	WU 30620	NA	HQ599439	HQ599454	NA	HQ599377	Voglmayr et al. (2011)
*Massarinaeburnea*	CBS 473.64	NA	GU301840	GU296170	GU371732	GU349040	Schoch et al. (2009)
*Massariosphaeriagrandispora*	CBS 613.86	NA	GU301842	GU296172	GU371725	GU349036	Schoch et al. (2009)
*Melogrammacampylosporum*	MBU (ET)	JF440978	NA	NA	NA	NA	Jaklitsch et al. (2016)
*Microdochiumphragmitis*	CBS 423.78 (ET)	MH861162	KP858948	NA	NA	NA	Vu et al. (2018)
*Microdochiumtrichocladiopsis*	CBS 623.77	KP858998	KP858934	NA	NA	NA	Hernandez et al. (2016)
*Monosporascuscannonballus*	FMR6682	NA	NA	AF340016	NA	NA	Collado et al. (2002)
*Neomassariafabacearum*	MFLUCC 16-1875 (HT)	NA	KX524145	KX524147	NA	NA	Mapook et al. (2016)
***Neomassariafabacearum***	**GMB0314**	**NA**	**ON4611373**	**ON461375**	**NA**	**ON505016**	**This study**
***Neomassariafabacearum***	**GMB0388**	**NA**	**ON505052**	**ON505050**	**NA**	**ON505019**	**This study**
*Neomassariaformosana*	NTUCC 17-007	NA	MH714756	MH714759	NA	NA	Ariyaw et al. (2018)
*Neomassariahongheensis*	KUMCC 21-0344 (HT)	NA	OL423113	OL423115	NA	NA	Yang et al. (2022)
*Neoroussoellabambusae*	MFLUCC 11-0124	KJ474827	KJ474839	NA	KJ474856	KJ474848	Liu et al. (2014)
*Neoroussoellaheveae*	MFLUCC 17-1983	MH590693	MH590689	NA	NA	NA	Senwanna et al. (2018)
*Neoroussoellasolani*	CPC 26331	KX228261	KX228312	NA	NA	NA	Crous et al. (2013)
*Neottiosporinapaspali*	CBS 331.37	NA	EU754172	EU754073	GU371779	GU349079	Gruyter et al. (2009)
*Oxydothiscalamicola*	MFLUCC 14-1165 (ET)	NA	KY206761	KY206767	NA	NA	Konta et al. (2016)
*Oxydothiscyrtostachicola*	FIH 151	DQ660334	DQ660337	NA	NA	NA	Hidayat et al. (2006)
***Oxydothisfortunei***	**GMB0315 (HT)**	**ON479893**	**ON479894**	**NA**	**NA**	**NA**	**This study**
***Oxydothisfortunei***	**GMB0389**	**ON510944**	**ON510945**	**NA**	**NA**	**NA**	**This study**
*Oxydothisinaequalis*	FIH 018	DQ660336	DQ660339	NA	NA	NA	Hidayat et al. (2006)
*Oxydothismetroxylonicola*	MFLUCC 15-0281 (ET)	KY206774	KY206763	KY206769	NA	NA	Konta et al. (2016)
*Oxydothispalmicola*	MFLUCC 15-0806 (ET)	KY206776	KY206765	KY206771	NA	NA	Konta et al. (2016)
*Oxydothisphoenicis*	MFLUCC 18-0270 (ET)	MK088066	MK088062	NA	NA	NA	Unpublished
*Oxydothisrhapidicola*	MFLUCC 14-0616 (ET)	NA	KY206766	KY206772	NA	NA	Konta et al. (2016)
*Paramassariasamaneae*	HKAS 102338	NA	NG068281	NG067686	NA	MK105748	Samarak and Hyde (2019)
*Pararoussoellamangrovei*	MFLU 17-1542 (HT)	MH025951	MH023318	NA	MH028250	MH028246	Wanasinghe et al. (2018)
*Pararoussoellamukdahanensis*	MFLU 11-0237 (HT)	NR155722	NA	NA	NA	NA	Dai et al.(2016)
*Pararoussoellarosarum*	MFLU 0654 (HT)	NR_157529	NG_059872	NA	NA	NA	Wanasinghe et al. (2018)
*Parathyridariapercutanea*	CBS 868.95	KF322118	KF366449	NA	KF366452	KF407987	Ahmed et al. (2014)
*Parathyridariaramulicola*	CBS 141479 (HT)	NR_147657	NA	NG_061254	KX650584	KX650536	Jaklitsch et al. (2016)
*Parathyridariarobiniae*	MFLUCC 14-1119 (HT)	KY511142	KY511141	NA	NA	KY549682	Unpublished
*Pestalotiopsistheae*	SAJ-0021 (ET)	JN943623	JN940838	JN940785	NA	NA	Unpublished
*Phialemoniumatrogriseum*	CBS 604.67	HE599384	HQ231981	NA	NA	NA	Summerbell et al. (2011)
*Pseudomassariachondrospora*	It 1200	KR092790	KR092779	NA	NA	NA	Senanayake et al. (2015)
*Pseudomassariachondrospora*	PC1 (ET)	JF440982	NA	NA	NA	NA	Jaklitsch et al. (2016)
*Pseudoneoconiothyriumeuonymi*	CBS:143426 (HT)	MH107915	MH107961	NA	MH108007	NA	Crous et al. (2018)
*Pseudoneoconiothyriumrosae*	MFLU 18-0117 (HT)	NR_157523	NG_059868	NA	NA	NA	Wanasinghe et al. (2018)
*Pseudoroussoellaelaeicola*	MFLUCC 15-15-0276a	MH742329	MH742326	NA	–	–	Unpublished
*Requienellaaquatic*	MFLUCC 18-1040 (HT)	NR_171975	NG_073797	NA	NA	NA	Unpublished
*Requienellachiangraina*	MFLUCC 10-0556 (HT)	NR_155712	NG_059510	NA	NA	NA	Liu et al. (2014)
*Requienelladoimaesalongensis*	MFLUCC 14-0584 (HT)	NR_165856	NG_068241	NA	KY678394	KY651249	Thambugala et al. (2017)
*Requienellaguttulata*	MFLUCC 20-0102 (HT)	NR_172428	NG_075383	NA	NA	NA	Zhang et al. (2020)
*Requienellahysterioides*	MAFF 239636	NA	AB524621	AB524480	AB539101	AB539114	Schoch et al. (2009)
*Requienellahysterioides*	CBS 546.94	MH862484	MH874129	NA	KF443392	KF443399	Vu et al. (2018)
*Requienellaintermedia*	CBS 170.96	KF443407	KF443382	NA	KF443394	KF443398	Ahmed et al. (2014)
*Requienellajapanensis*	MAFF 239636 (HT)	NR_155713	NA	NA	NA	NA	Liu et al. (2014)
*Requienellakunmingensis*	HKAS 101773 (HT)	MH453491	MH453487	NA	MH453484	MH453480	Unpublished
*Requienellamagnatum*	MFLUCC 15-0185 (HT)	NA	KT281980	NA	NA	NA	Unpublished
*Requienellamargidorensis*	MUT 5329 (HT)	NR169906	MN556322	NA	MN605917	MN605897	Poli et al. (2020)
*Requienellamediterranea*	MUT5369 (HT)	KU314947	MN556324	NA	MN605919	MN605899	Poli et al. (2020)
*Requienellamexicana*	CPC 25355 (HT)	KT950848	KT950862	NA	NA	NA	Crous et al. (2014)
***Requienellabambusarum***	**GMB0316 (HT)**	**ON479891**	**ON479892**	**NA**	**ON505011**	**ON505015**	**This study**
***Requienellabambusarum***	**GMB0390**	**ON505055**	**ON505051**	**NA**	**ON505012**	**ON505017**	**This study**
*Requienellaneopustulans*	MFLUCC 11-0609 (HT)	KJ474833	KJ474841	NA	NA	KJ474850	Liu et al. (2014)
*Requienellanitidula*	MFLUCC 11-0634	KJ474834	KJ474842	NA	KJ474858	KJ474851	Liu et al. (2014)
*Requienellapadinae*	MUT 5503 (HT)	NA	MN556327	NA	MN605922	MN605902	Poli et al. (2020)
*Requienellapseudohysterioides*	GMBC0009 (HT)	MW881445	MW881451	NA	MW883345	NA	Unpublished
*Requienellapustulans*	KT 1709	NA	AB524623	NA	AB539103	AB539116	Tanaka et al. (2009)
*Requienellaseminuda*	RS12	KT949912	NA	NA	NA	NA	Jaklitsch et al. (2016)
*Requienellaseminuda*	RS13	KT949913	NA	NA	NA	NA	Jaklitsch et al. (2016)
*Requienellasiamensis*	MFLUCC 0149 (HT)	KJ474837	KJ474845	NA	KJ474861	KJ474854	Liu et al. (2014)
***Requienellasiamensis***	**GMB0317**	**ON4617749**	**ON461896**	**NA**	**ON505010**	**ON505014**	**This study**
***Requienellasiamensis***	**GMB0391**	**ON505054**	**ON505053**	**NA**	**ON505013**	**ON505018**	**This study**
*Requienellathailandica*	MFLUCC 0621 (HT)	KJ474838	KJ474846	NA	NA	NA	Liu et al. (2014)
*Requienellatosaensis*	KT 1659	NA	AB524625	NA	AB539104	AB539117	Tanaka et al. (2009)
*Requienellatuberculata*	MFLUCC 0854 (HT)	KU940132	KU863121	NA	NA	KU940199	Dai et al. (2016)
*Requienellaverrucispora*	CBS 125434 (HT)	KJ474832	NA	NA	NA	NA	Liu et al. (2014)
*Requienellayunnanensis*	HKAS 101762	MH453492	MH453488	NA	NA	MH453481	Unpublished
*Robillardasessilis*	CBS 114312 (ET)	KR873256	KR873284	NA	NA	NA	Crous et al. (2014)
*Robillardaterrae*	CBS 587.71	KJ710484	KJ710459	NA	NA	NA	Crous et al. (2014)
*Roussoellascabrispora*	MFLUCC 14-0582	KY026583	KY000660	NA	NA	NA	Unpublished
*Roussoellopsismacrospora*	MFLUCC 12-0005	NA	KJ474847	NA	KJ474862	KJ474855	Liu et al. (2014)
*Seiridiumphylicae*	CPC 19962	KC005785	KC005807	NA	NA	NA	Crous et al. (2012)
*Seynesiaerumpens*	SMH 1291	NA	AF279410	AF279409	NA	NA	Bhattacharya et al. (2000)
*Subramaniomycesfusisaprophyticus*	CBS 418.95	EU040241	NA	NA	NA	NA	Crous et al. (2007)
*Thyridariaacaciae*	CBS:138873	KP004469	KP004497	NA	NA	NA	Crous et al. (2014)
*Thyridariabroussonetiae*	CBS 121895	KX650567	NA	NA	KX650585	KX650538	Jaklitsch et al. (2016)
*Thyridariellamahakoshae*	NFCCl 4215	MG020435	MG020438	NA	MG020446	MG023140	Devadatha et al. (2018)
*Thyridariellamangrovei*	NFCCl 4213	MG020434	MG020437	NA	MG020445	MG020443	Devadatha et al. (2018)
*Torulaherbarum*	CBS 111855	KF443409	KF443386	NA	KF443396	KF443403	Ahmed et al. (2014)
*Trematosphaeriapertusa*	CBS 122371	NA	GU301876	GU348999	GU371801	GU349085	Schoch et al. (2009)
*Vialaeamangiferae*	MFLUCC 12-0808	KF724974	KF724975	NA	NA	NA	Senanayake et al. (2014)
*Vialaeaminutella*	BRIP 56959 (ET)	KC181926	KC181924	NA	NA	NA	McTaggart et al. (2013)
*Xylariahypoxylon*	CBS 122620 (ET)	AM993141	NA	NA	NA	NA	Persoh et al. (2009)
*Xylariapolymorpha*	MUCL: 49904	FN689809	NA	NA	NA	NA	Fournier et al. (2011)
*Zopfiarhizophila*	CBS 207.26	NA	DQ384104	L76622	NA	NA	LoBuglio et al. (1996)

Notes: Type specimens are marked with HT (holotype), ET (epitype); NA: No sequence is available in GenBank; newly generated sequences are indicated in bold.

### ﻿Phylogenetic analysis

All sequences used for phylogenetic analysis were downloaded from the GenBank, based on published literature and the highest hit rate of ITS in the GenBank database. Sequence data for the construction of the phylogenetic trees are listed in Table 1. Single gene sequence alignments were generated with MAFFT v.7.110 (http://mafft.cbrc.jp/alignment/server/index.html, Katoh and Standley 2013) and multiple sequence alignments were edited manually when necessary in BioEdit v.7.0 (Hall 1999). ALTER (http://www.sing-group.org/ALTER/) was used to convert the file format (Alignment Transformation Envi-Ronment). The maximum likelihood analysis was carried out with GTR+G+I model of site substitution by using RAxML 8.2.12 BlackBox. Bayesian Inference (BI) analysis was performed with MrBayes v.3.2.7a (Huelsenbeck 2012). The branch support was evaluated with 1000 bootstrap replicates (Silvestro and Michalak 2012). Posterior probabilities (PP) were determined by Markov Chain Monte Carlo sampling (MCMC) in MrBayes v. 3.2.7a (Ronquist et al. 2012). Trees were visualized by FigTree v. 1.4.4, and additionally, layouts were done with Photoshop CS6. The alignments and respective phylogenetic trees were uploaded in TreeBASE (http://www.treebase.org. submission number: ID 29735; ID 29736; ID 29737).

### ﻿Abbreviations

**AFTOL-ID**: Assembling the Fungal Tree of Life; **ATCC**: American Type Culture Collection; **CBS**: Centraalbureau voor Schimmelcultures, Utrecht, The Netherlands; **CMW**: Tree Pathology Co-operative Program, Forestry and Agricultural Biotechnology Institute, University of Pretoria, South Africa; **CPC**: Culture collection of Pedro Crous, housed at the Westerdijk Fungal Biodiversity Institute; **GMB**: herbarium of Guizhou Medical University; **HKAS**: herbarium of Cryptogams Kunming Institute of Botany Academia Sinica, Chinese Academy of Sciences, Kunming, China; **HKUCC**: Hong Kong University Culture Collection; **ICMP**: International Collection of Microorganisms from Plants; **IMI**: CABI Bioscience UK Centre; **JK**: J. Kohlmeyer; **KT**: K. Tanaka; **KUMCC**: Kunming Institute of Botany Culture Collection, Chinese Science Academy, Kunming, China; **MAFF**: Ministry of Agriculture, Forestry and Fisheries, Japan; **MFLU**: Mae Fah Luang University Herbarium, Chiang Rai, Thailand; **MFLUCC**: Mae Fah Luang University Culture Collection, Chiang Rai, Thailand; **MUCL**: University Catholique de Louvain; **NFCCI**: National Fungal Culture Collection of India; **SMH**: Sabine M. Huhndorf; **WU**: Fungarium of the Department of Botany and Biodiversity Research, University of Vienna; **Others**: information not available.

## ﻿Results

### ﻿Phylogenetic analyses

Three phylogenetic trees for each genus and their related genera were provided.

**Figure 1.**
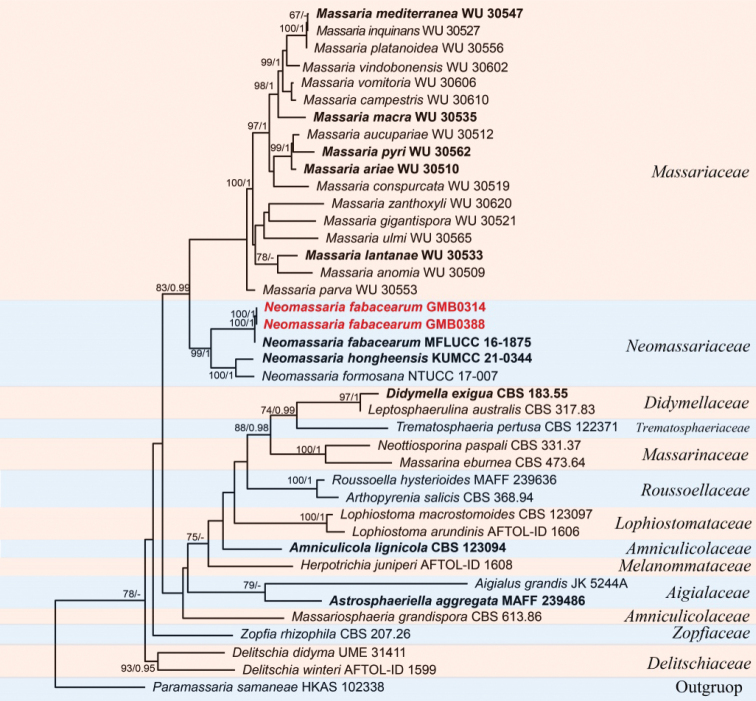
RAxML tree of *Neomassaria* and related genera obtained from the concatenated DNA sequence data of LSU, SSU and *tef*1 genes. Bootstrap support values for ML equal to or greater than 60% and BYPP equal to or greater than 0.95 are given above the nodes. The new collections are in red bold and type strains are in bold.

The dataset for Fig. 1 consists of 40 taxa for representative strains of species in Neomassariaceae, which has 1989 characters including gaps (SSU: 1–515, *tef*1:516–1192, LSU: 1193–1989). The best scoring likelihood tree was selected with a final ML optimization likelihood value of -23512.21. *Paramassariasamaneae* Samarak & K.D. Hyde (HKAS 102338) was selected as the outgroup taxon. Strain GMB0314 gathered with *N.fabacearum* with high statistical support (100% ML, 1.00 BYPP, Fig. 1).

**Figure 2.**
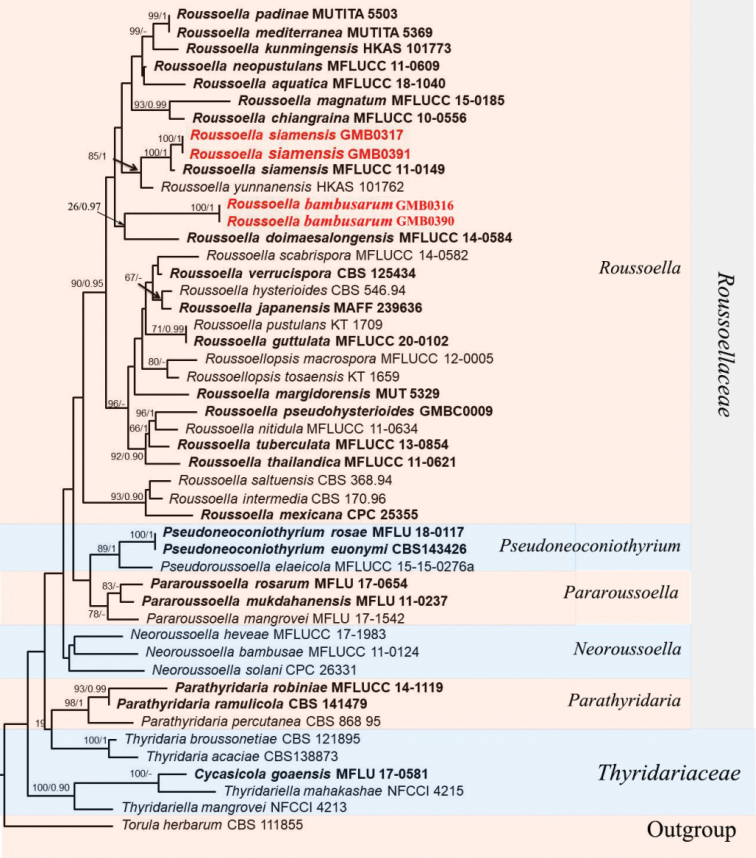
RAxML tree of *Roussoella* and related genera based on a combined ITS, LSU, *rpb*2 and *tef*1 sequences dataset. Bootstrap support values for ML equal to or greater than 60% and BYPP equal to or greater than 0.95 are given above the nodes. The new collections are in red bold, type strains are in bold.

The dataset for Fig. 2 consists of 46 taxa for representative strains of species in Roussoellaceae with 2330 characters, including gaps (ITS: 1–375, *tef*1: 376–1063, LSU: 1064–1592, *rpb*2: 1593–2330). The final ML optimization likelihood value of the best scoring likelihood was -16254.35. *Torulaherbarum* Link (CBS 111855) was selected as the outgroup taxon. Strains of the *R.bambusarum* formed a clade with *R.doimaesalongensis* Thambug. & K.D. Hyde with statistical support (26% ML, 0.97 BYPP). Strain GMB0317 gathered with *R.siamensis* Phook., Jian K. Liu & K.D. Hyde with high statistical support (100% ML, 1.00 BYPP, Fig. 2).

**Figure 3.**
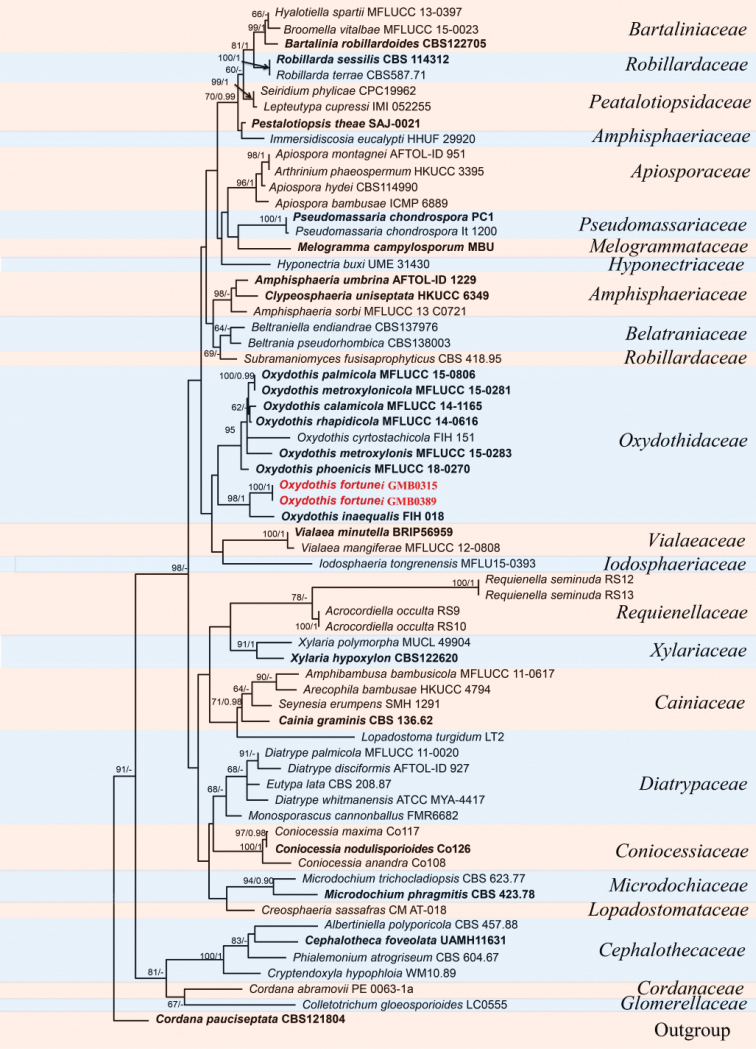
RAxML tree of *Oxydothis* and related genera based on a combined ITS, LSU and SSU sequences dataset. Bootstrap support values for ML equal to, or greater than, 60% and BYPP equal to or greater than 0.95 are given above the nodes. The new collections are in red bold and ex-type strains are in bold.

The alignment for Fig. 3 consists of 66 taxa for representative strains of species in Oxydothidaceae including outgroup taxa with 1630 characters (ITS: 1–307, LSU: 308–1089, SSU: 1090–1630). The best scoring likelihood tree was selected with a final ML optimization likelihood value of -19975.73. *Cordanapauciseptata* Preuss (CBS 121804) was selected as the outgroup taxon. Our strains of the new species *O.fortunei* are from a distinct clade with *O.inaequalis* Hidayat et al. (98% ML, 1 BYPP, Fig. 3).

### ﻿Taxonomy

The four species in this study were *Neomassariafabacearum*, *Roussoellabambusarum*, *Roussoellasiamensis*, *Oxydothisfortunei*. *Neomassaria* and *Roussoella* is a genus of ascomycete fungi in the order Pleosporales. *Oxydothis* is a genus of ascomycete fungi in the order *Xylariales*.

#### ﻿ Neomassaria fabacearum

Taxon classificationFungiPleosporalesMassariaceae

Mapook, Camporesi & K.D. Hyde, Fungal Diversity 80: 77 (2016)

FD8BE88D-0A16-5CDF-AC94-3A8BD25C2783

 552274

Fig. 4

##### Descriptions.

see Hyde et al. (2016).

##### Specimens examined.

China, Guizhou Province, the campus of Guizhou Medical University (26°24'34.02"N, 106°45'16.22"E), on bamboo, 12 December 2021. Altitude: 1145 m, H.M. Hu, 2021GYHS23 (GMB0314; KUN-HKAS 123429; living culture GMBC0314).

**Figure 4.**
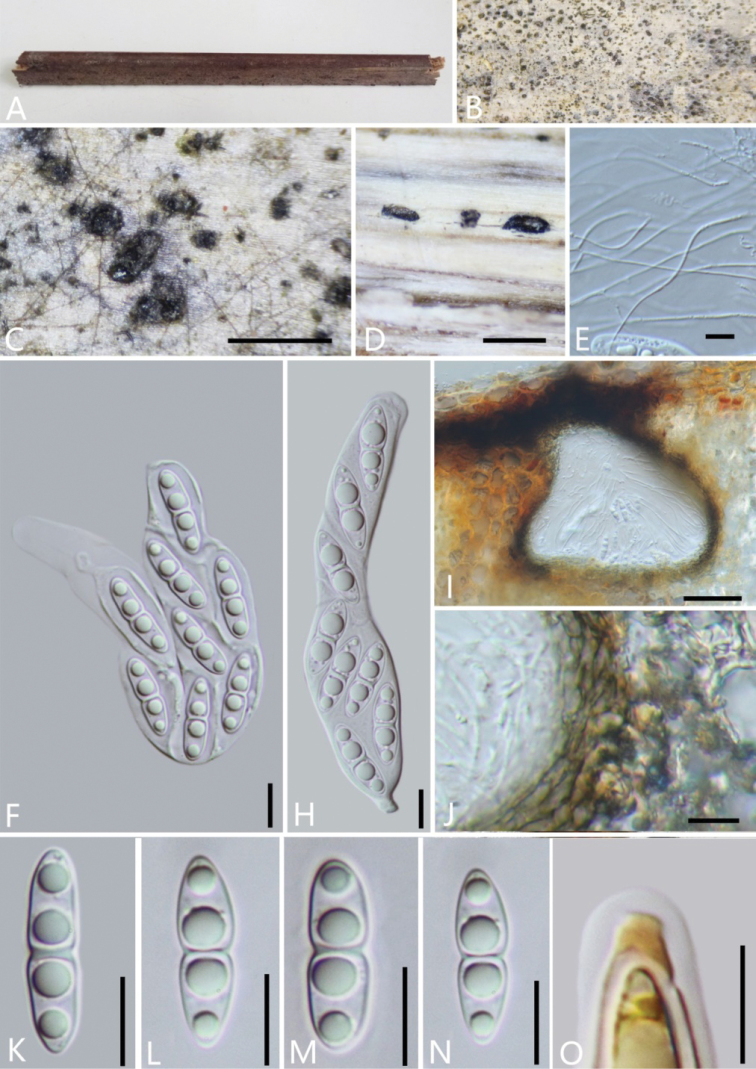
*Neomassariafabacearum* (GMB0314) **A** stromata on host substrate **B, C** appearance of ascomata on substrate **D** cross section of ascomata **E** pseudoparaphyses **F, H** asci **I** longitudinal section of an ascoma **J** peridium **K–N** ascospores **O** apical apparatus (stained in Melzer’s Reagent). Scale bars: 0.5 mm (**C–D**); 10 μm (**E–H, K–O**); 50 μm (**I, J**).

##### Other material examined.

China, Guizhou Province, the campus of Guizhou Medical University (26°24'34.01"N, 106°45'09.24"E), on bamboo, 12 December 2021. Altitude: 1135 m, H.M. Hu, 2021GYHS28 (GMB0388, living culture GMBC0388).

##### Notes.

There are three *Neomassaria* species documented in Index Fungorum (accession date: May 1, 2022). Type species of *N.fabacearum* was originally described from Italy (Hyde et al. 2016). Subsequently, *N.formosana*, and *N.hongheensis* were introduced from Taiwan and Yunnan in China, respectively (Ariyawansa et al. 2018; Yang et al. 2022). The ascospore dimension of *N.fabacearum* is between those of *N.formosana* (20–30 × 3–7 μm) and *N.hongheensis* (14–17 × 4–8 μm) (Hyde et al. 2016; Ariyawansa et al. 2018; Yang et al. 2022). Phylogenetic analyses of the combined SSU, LSU and *tef*1 sequences dataset shows that new collections gather with *N.fabacearum* (MFLU 16–1875), the type specimen, with the high support (100% ML, 1 BYPP; Fig. 1). Morphologically, the features of GMB0314 are consistent with those of *N.fabacearum* (Hyde et al. 2016). *Neomassariafabacearum* was first introduced to the China.

#### ﻿ Roussoella bambusarum

Taxon classificationFungiPleosporalesThyridariaceae

H. M. Hu & Q. R. Li sp. nov.

156BC038-8386-503B-8746-A81DE1550254

 844142

Fig. 5

##### Holotype.

GMB0316.

##### Etymology.

In reference to the host, *Bambusabambusarum* (Lour.) Raeusch. ex Schult. ‘Fernleaf’ R. A. Young

##### Description.

***Saprobic*** on decaying culms of *B.bambusarum*. **Sexual morp: *Ascostromata*** 111–146 μm high, 460–560 μm diam., (x̄ = 123 × 539 μm, n = 30), immersed under a clypeus, solitary or scattered, raised hemispherical or dome-shaped on host epidermis, black, coriaceous, glabrous, uni-loculate. ***Locules*** 335–414 μm diam., 128–212 μm high, immersed within ascostromata, black, globose to subglobose. ***Ostioles*** with minute papillate. ***Peridium*** 19–34 μm thick, composed of dark brown thin-walled cells of *textura angularis*. ***Hamathecium*** comprised of 1–2 μm wide, numerous, septate, branched, anastomosing, filiform, hyaline, pseudoparaphyses. ***Asci*** 120–143 × 8–12 μm (x̄ = 134 × 10 μm, n = 30), 8-spored, bitunicate, cylindrical, curved, short pedicellate with knob-like pedicel, apically rounded with an indistinct ocular chamber. ***Ascospores*** 14–20 × 6–7 μm (x̄ = 17.6 × 6.7 μm, n = 30), dark brown to brown, 1-seriate, sometimes overlapping, 2-celled, constricted at the septum, ellipsoidal to fusiform, straight, rough-walled, guttulate, conically rounded ends, with longitudinal striations. **Asexual morph**: Undetermined.

**Figure 5.**
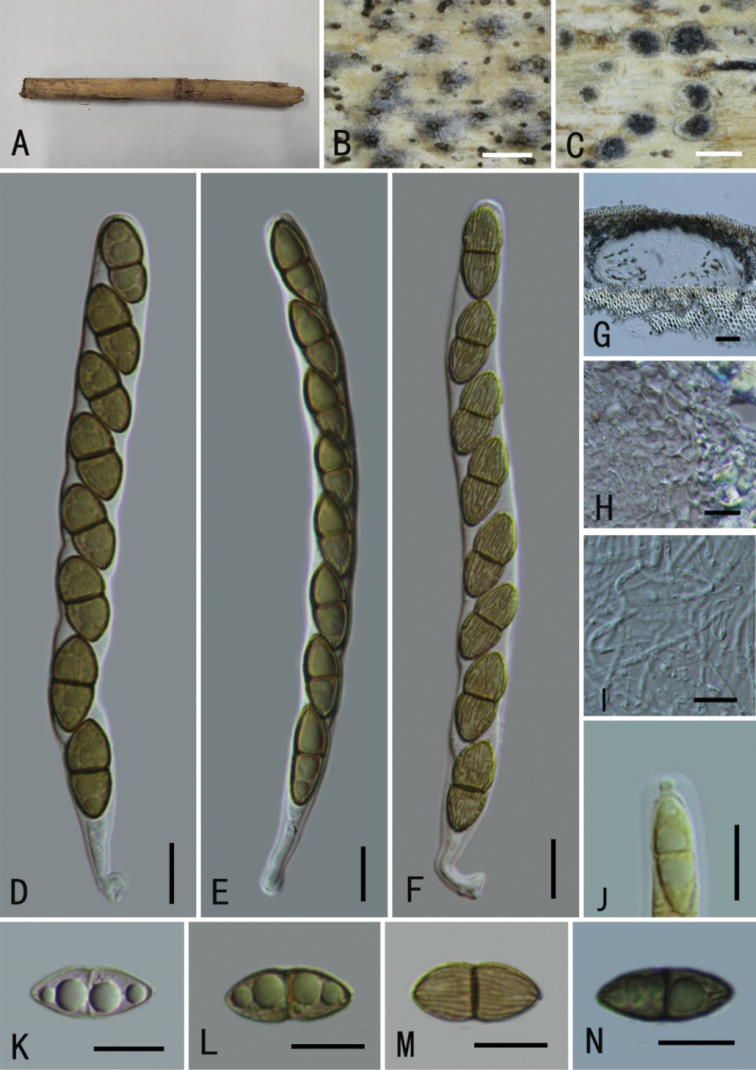
*Roussoellabambusarum* (Holotype, GMB0316) **A** stromata on host substrate **B** ascostromata on bamboo culm **C** cross-section of ascostromata **D–F** asci **G** longitudinal section of ascostromata **H** peridium **I** pseudoparaphyses **J** apical apparatus (stained in Melzer’s Reagent) **K–N** ascospores. Scale bars: 0.5 mm (**B–C**); 10 μm (**D–F, H–N**); 50 μm (**G**).

##### Culture characters.

Ascospores germinated on PDA within 24 hours at 25 °C, colonies are reaching 5 cm diam. The colony on the surface is white, grey, circular, flocculent, dense, cottony mycelium, colony reverse is white and gray, white in the middle. Not sporulating on OA nor on PDA.

##### Specimens examined.

China, Guizhou Province, Guiyang Huaxi National Urban Wetland Park (26°2'2.34"N, 106°34'16.22"E), on decaying culms of *B.bambusarum*, 12 October 2021. Altitude: 1130 m, Y.P Wu and H.M Hu, 2021 HXGY01 (GMB0316, holotype; KUN-HKAS 123431, isotype; GMBC0316, ex-type living culture).

##### Other examined material.

China, Guizhou Province, Guiyang Huaxi National Urban Wetland Park (26°10'44.13"N, 106°43'13.12"E), on decaying culms of *B.bambusarum*, 15 October 2021. Altitude: 1201 m, Y.P Wu and H.M Hu, 2021 HXGY55 (GMB0390; GMBC0390, living culture).

##### Notes.

Morphologically, *Roussoellabambusarum* is similar to *R.thailandica* D.Q. Dai et al., but differs from the latter by having larger ascospores (17.6 × 6.7 μm vs. 14.5 × 5.5 μm), larger upper cells, occasionally curve, narrowly at both ends, with irregular longitudinal striations. (Liu et al. 2014). Phylogenetic analysis showed that *R.bambusarum* and *R.doimaesalongensis* Thambug. & K.D. Hyde were clustered together (26% ML, 0.97 BYPP; Fig. 2) (Thambugala et al. 2017).

#### ﻿ Roussoella siamensis

Taxon classificationFungiPleosporalesThyridariaceae

Phook., Jian K. Liu & K.D. Hyde, Phytotaxa 181(1): 18 (2014)

E1D19AD1-2746-521F-B7AE-A70A8BB50D3C

 550665

Fig. 6

##### Descriptions.

see Liu et al. (2014).

##### Specimens examined.

China, Guizhou Province, Guiyang Huaxi National Urban Wetland Park (26°2'23.04"N, 106°34'16.22"E) on decaying culms of *B.bambusarum*, 12 October 2021. Altitude: 1130 m, Y.P. Wu and H.M. Hu, 2021 HXGY03 (GMB0317; living culture GMBC0317).

**Figure 6.**
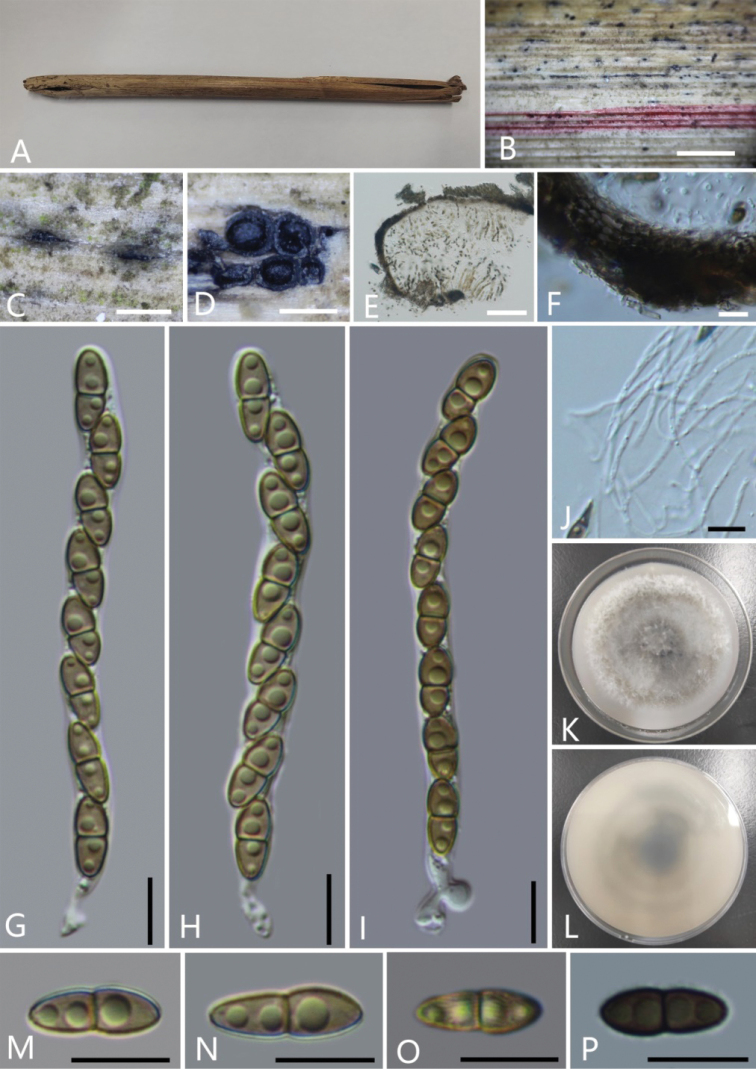
*Roussoellasiamensis* (GMB0317) **A** stromata on host substrate **B, C** ascostromata on bamboo culm **D** cross-section of ascostromata **E** Longitudinal section of ascostromata **F** peridium **G–I** asci **J** pseudoparaphyses **K–L** culture on PDA**M–P** ascospores Scale bars: 0.5 mm (**C–D**); 50 μm (**E**); 10 μm (**F–J, M–P**).

##### Other material examined.

China, Guizhou Province, Guiyang Huaxi National Urban Wetland Park (26°2'10.10"N, 106°34'16.10"E) on decaying culms of *B.bambusarum*, 15 October 2021. Altitude: 1145 m, Y.P. Wu and H.M. Hu, 2021 HXGY70 (GMB0391; living culture GMBC0391).

##### Notes.

Phylogenetic analyses of the alignment combining ITS, LSU, *rpb*2 and *tef*1 show that GMB0317 cluster with *R.siamensis* (MFLU 13-0639) with the high support value (100% ML, 1 BYPP; Fig. 2). Characteristics of GMB0317 are consistent with those of *R.siamensis*, which was originally introduced from decaying bamboo culms in Thailand (Liu et al. 2014) This species was first found in China.

#### ﻿ Oxydothis fortunei

Taxon classificationFungiXylarialesAmphisphaeriaceae

H. M. Hu & Q. R. Li sp. nov.

ACF12041-230E-51CB-841D-3E7882153FA4

 844141

Fig. 7

##### Holotype.

GMB0315.

##### Etymology.

In reference to the host, *Trachycarpusfortunei* (Hook.) H. Wendl.

##### Description.

***Saprobic*** on surface of culms of *T.fortunei*. **Sexual morph: *Ascomata*** 205–317 μm diam. (x̄ = 261 μm, n = 30), solitary or aggregated in groups, immersed, forming slightly raised as blistering areas on the host surface, long axis horizontal to that of the host, 18–41 μm high × 155–207 μm broad, in transverse section, ellipsoid, ostiolate, coriaceous, black, flat. ***Peridium*** 24–27 μm thick, composed of 2–3 several layers of flattened, light-brown cells. ***Asci*** 108–121× 9–14 μm (x̄ = 114 × 12 μm, n = 20), 8-spored, unitunicate, cylindrical, mostly straight, pedicellate, with a J-, subapical apparatus, 4.2–4.9 μm high, 5.5–6.8 μm diam. ***Ascospores*** 56–72 μm × 3–4 μm (x̄ = 66 × 3.3 μm, n = 30), fusiform, hyaline, obliquely 1–2-seriate, tapering gradually from the center to the ends, with multi-guttules in each cell, pointed processes. **Asexual morph**: Undetermined.

**Figure 7.**
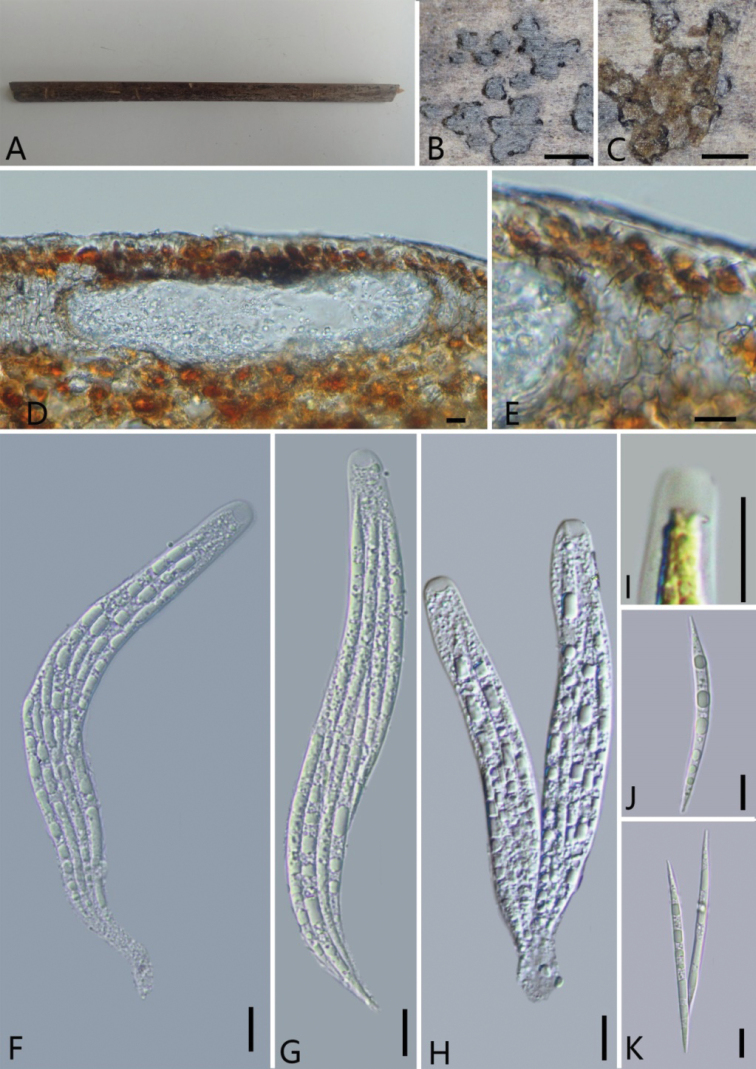
*Oxydothisfortunei* (Holotype, GMB0315) **A** stromata on host substrate **B** close-up of ascomata **C** cross-section of the ascomata **D** longitudinal section of an ascoma **E** peridium **F–H** asci **I** apical apparatus (stained in Melzer’s Reagent) **J, K** ascospores. Scale bars: 0.5 mm (**B–C**); 10 μm (**D–K).**

##### Culture characteristics.

Ascospores germinated on PDA within 24 hours at 25 °C, colonies are reaching 4.5 cm diam. circular, transparent, thin, colony reverse is same. Not sporulating on OA nor on PDA.

##### Specimen examined.

China Guizhou Province, Long gong scenic spot (26°04'35.02"N, 105°52'15.04"E), on surface of culms of *T.fortunei*, 5 December 2021. Altitude: 1120m, Q.R. Li and X. Xu, 2021 LG9 (GMB0315, holotype; KUN-HKAS 123430, isotype; ex-type living culture GMBC0315).

##### Other examined material.

China, Guizhou Province, Long gong scenic spot (26°04'47.41"N, 105°31'10.34"E), on surface of culms of palm, 7 December 2021. Altitude: 1095m, Q.R. Li and X. Xu, 2021 LG15 (GMB0389; living culture GMBC0389).

##### Notes.

*Oxydothisfortunei* is morphologically similar to *O.nonamyloidea* K.D. Hyde and *O.rhapidicola* S. Konta & K.D. Hyde in the shape of ascospores (Hyde 1994; Hidayat et al. 2006; Konta et al. 2016). However, the ascospores of *O.fortunei* (56–72 × 3–4 μm) are shorter than those of *O.nonamyloidea* (94–115 × 3.5–4.5 μm) and *O.rhapidicola* (47–50 × 3–5 μm). Moreover, it is distinguished from *O.rhapidicola* since the latter has a blue slit-like ascus subapical apparatus in Melzer’s reagent (Konta et al. 2016). *Oxydothisfortunei* showed the close kinship to *O.inaequalis* (100% ML, 1 BYPP; Fig. 3). However, *O.fortunei* differs from *O.inaequalis* by its shape of the ascospores, and the J- ascus subapical apparatus as well as the smaller ascospores (56–72 × 2.9–3.9 μm vs. 78–100 × 5–6 μm) (Hidayat et al. 2006).

## ﻿Discussion

In this study, two new species and two new records associated with bamboo and palm were introduced based on phylogenetic relationships of combined ITS, LSU, SSU, *rpb*2 and *tef*1 sequences and morphological evidences.

There are a large number of fungi associated with bamboo and palm in China (Hyde et al. 2002; Phukhamsakda et al. 2022). Studies on the diversity of bamboo and palm fungi can be of economic significance and of academic value (Arnold and Lewis 2005). According to statistics, there are nearly 500 bamboo species distributed in 37 genera in China, which play an important role in human life, such as in the fields of architecture, production tools, artwork, and landscaping, etc. (Zhao and Wei 2018). In China, palms are mainly used for ornamental purposes in landscape gardens (Fetouh et al. 2018). About 2,450 species of palm plants were documented in the world, belonging to 183 genera (Qureshimatva et al. 2018). The rich and diverse ecosystems composed of these bamboo and palm resources provide good habitats for fungi to survive, creating the diversity of fungal species (Cheek et al. 2020). There are 75 genera and 189 fungal species on bamboo that have been reported in mainland China, and 79 species and 58 genera of bamboo fungi that have been reported in Hong Kong (Yong et al. 2009; Shukla et al. 2016). Many species of *Roussoella* have been introduced from the bamboo (Liu et al. 2014). New collections of *Roussoella* also were saprophyte on bamboo. Most species of *Oxydothis* were discovered on palm including *O.fortunei* (Konta et al. 2016). This is the first introduction of *Neomassaria* species associated on bamboo (Ariyawansa et al. 2018; Yang et al. 2022). In this study, four microfungi were introduced, which enriches the diversity of fungi on bamboo and palm in China. Meanwhile, all those four species are saprophyte on and accelerates the decay of bamboo or palm. As an ideal growth substrate for fungi, bamboo fungi are rich in species, and there are a large number of fungi to be discovered.

## Supplementary Material

XML Treatment for Neomassaria fabacearum

XML Treatment for Roussoella bambusarum

XML Treatment for Roussoella siamensis

XML Treatment for Oxydothis fortunei
